# Chimpanzees create and modify probe tools functionally: A study with zoo-housed chimpanzees

**DOI:** 10.1002/ajp.22319

**Published:** 2014-09-12

**Authors:** Lydia M Hopper, Claudio Tennie, Stephen R Ross, Elizabeth V Lonsdorf

**Affiliations:** 1 Lester E Fisher Center for the Study and Conservation of Apes, Lincoln Park ZooChicago, Illinois; 2 School of Psychology, University of BirminghamBirmingham, United Kingdom; 3 Department of Psychology, Franklin and Marshall CollegeLancaster, Pennsylvania

**Keywords:** chimpanzee, tool use, probe tool, tool modification, learning

## Abstract

Chimpanzees (*Pan troglodytes*) use tools to probe for out-of-reach food, both in the wild and in captivity. Beyond gathering appropriately-sized materials to create tools, chimpanzees also perform secondary modifications in order to create an optimized tool. In this study, we recorded the behavior of a group of zoo-housed chimpanzees when presented with opportunities to use tools to probe for liquid foods in an artificial termite mound within their enclosure. Previous research with this group of chimpanzees has shown that they are proficient at gathering materials from within their environment in order to create tools to probe for the liquid food within the artificial mound. Extending beyond this basic question, we first asked whether they only made and modified probe tools when it was appropriate to do so (i.e. when the mound was baited with food). Second, by collecting continuous data on their behavior, we also asked whether the chimpanzees first (intentionally) modified their tools prior to probing for food or whether such modifications occurred after tool use, possibly as a by-product of chewing and eating the food from the tools. Following our predictions, we found that tool modification predicted tool use; the chimpanzees began using their tools within a short delay of creating and modifying them, and the chimpanzees performed more tool modifying behaviors when food was available than when they could not gain food through the use of probe tools. We also discuss our results in terms of the chimpanzees’ acquisition of the skills, and their flexibility of tool use and learning. Am. J. Primatol. 77:162–170, 2015. © 2014 The Authors. *American Journal of Primatology* Published by Wiley Periodicals Inc.

## INTRODUCTION

There are numerous accounts of chimpanzees (*Pan troglodytes*) using tools to probe for out-of-reach food, both in the wild and in captivity [e.g., Boesch & Boesch, 1990; Goodall, 1986; McGrew et al., 1979, see Sanz et al., 2013 and Shumaker et al., 2011 for reviews]. Furthermore, such probing behavior by chimpanzees is not restricted to a single realm; chimpanzees have been observed to probe using a variety of techniques and for a number of foodstuffs, including when termite fishing [e.g., Lonsdorf et al., 2004], ant dipping [e.g., Sugiyama et al., 1988], and honey dipping [e.g., Boesch et al., 2009]. Beyond ecological studies of ape tool use in the wild [e.g., Möbius et al., 2008], research with captive great apes has also begun to elucidate their understanding of the physical and causal properties of tools [Horner & Whiten, 2007; Mulcahy et al., 2013; Mulcahy & Call, 2006; Mulcahy & Schubiger, 2014; Seed et al., 2012]. Such research has revealed that in certain circumstances chimpanzees will select tools that have the appropriate physical properties required for the task [e.g., intact, not broken: Seed et al., 2012] and can modify tools in order to make them suited to the specific functions of the task they are presented with [e.g., Bania et al., 2009]; skills that appear to vary across individuals by age and rearing history.

In addition to gathering appropriately-sized materials, chimpanzees also perform secondary modifications in order to create an optimal tool [McGrew, 2013]. To create tools, chimpanzees can modify available substrates in one of four ways [Shumaker et al., 2011]: (1) by ‘detaching’ the tool material [e.g. pulling leaves from braches to then use as water sponges, Watts, 2008], (2) by ‘combining’ objects [e.g. to extend the length of a tool, Price et al., 2009], (3) by ‘reducing’ the material [e.g. removing side branches or thorns from a stalk, Sugiyama & Koman, 1979], and/or (4) by ‘reshaping’ the material to create a novel tool [e.g. when leaf-folding, Sousa et al., 2009]. The manner by which chimpanzees manufacture tools not only means that they are capable of modifying naturally-occurring substrates, but also that they can create different kinds of tools for specific purposes [Bania et al., 2009; Bermejo & Illera, 1999]. This is important because, although tool use is ubiquitous among a number of species, the manufacture and modification of tools is far less common [Beck et al., 2011; Byrne et al., 2013]. Tool modification, therefore, provides an avenue of research for testing ape physical cognition and causal understanding [Emery & Clayon, 2009; Schrauf & Call, 2009].

Considering the modification of probe tools, wild chimpanzees at some study sites fray the ends of their tool to apparently aid termite fishing [e.g. Bermejo & Illera, 1999; Sanz et al., 2009] and honey collection [e.g. Boesch et al., 2009]. Chimpanzees at Loango National Park, Gabon, for example, create multiple tool types for honey extraction, but only one, the ‘honey collection tool’, has been found to be frayed at one end [Boesch et al., 2009]. Boesch and colleagues inferred that honey collection tools were used by the chimpanzees to actively extract honey, while the other four tool types were used to break open beehives. If these inferences are correct, honey collection tools, are akin to modified probe tools used by chimpanzees in other wild communities to ‘bee probe’ [Fowler & Sommer, 2007] and ‘fluid dip’ [Sanz & Morgan, 2009]. Without observing the chimpanzees’ behavior, it cannot be determined whether they modify their tools (intentionally) prior to use. At the Goualougo Triangle, Republic of Congo, however, tool fraying has been observed directly [Sanz & Morgan, 2007] and Sanz et al. [2009] reported that “brush manufacture occurred prior to contact with the termite nest in 96 % of observations” [p. 294]. This suggests that tool modification may be intentional, and does not simply arise from the apes using the tools.

Although it has been previously shown that captive chimpanzees use probe tools proficiently [e.g. Hopper et al., 2007; Lonsdorf et al., 2009; Visalberghi et al., 1995], less is known about probe tool manufacture and modification. It has been reported, for example, that chimpanzees can combine tools, to increase their length, in order to obtain out-of-reach food items [Price et al., 2009] and can remove artificial ‘side branches’ from tools in order to make functional probe tools [Bania et al., 2009]. In our study, we wished to assess whether a group of chimpanzees would show evidence for tool modification and, if they did modify tools, whether they only made probe tools when it was appropriate to do so. Bania et al. [2009] showed that chimpanzees would modify tools in response to the physical properties of different experimental tasks they were presented with. In contrast, we evaluated whether chimpanzees would only modify and use tools when food was available. To test this, we presented a group of unenculturated zoo-housed chimpanzees that, to the best of our and the keepers’ knowledge, were naïve to using probe tools for extractive foraging at the start of the study, with an artificial termite mound first when not baited (baseline phase) and later when baited with food (test phase). These data were collected at the same time as those reported by Lonsdorf et al. [2009], who showed that this group of chimpanzees quickly developed probing skills when presented with the baited mound. However, our focus was tool-modification behaviors rather than simply tool use. If tool modification was functional, we predicted that the chimpanzees should (1) modify their tools prior to probing with them, (2) begin using the tool within a short delay of creating it, and (3) show more tool modifying behaviors when the artificial mound was baited with food than when it contained no food rewards.

## METHODS

### Subjects and Testing Environment

The subjects were seven captive-born chimpanzees housed together at the Regenstein Center for African Apes at the Lincoln Park Zoo (Chicago, Illinois). The group was comprised of three males and four females, with an average age of 10 years (range: 4–19 years). The chimpanzees inhabited an expansive indoor/outdoor exhibit that included climbing structures and deep-mulch bedding and an off-exhibit holding area. The indoor exhibit space also featured an artificial termite mound [Bonnie et al., 2012; Lonsdorf et al., 2009]. Throughout the study, the chimpanzees had outdoor access when weather conditions were appropriate (>5°C). Fresh produce and primate chow were scattered twice daily throughout their exhibits.

This study was approved by the Lincoln Park Zoo Research Committee, which is the governing body for all animal research at the institution. No social group manipulations occurred as the result of this project. Food substances, amount, and frequency were reviewed and approved by veterinary and nutrition staff prior to the start of the project. No modifications were made to standard animal care routines. This research adhered to legal requirements in the United States of America and to the American Society of Primatologists’ Principles for the Ethical Treatment of Nonhuman Primates.

### Apparatus and Tools

**Figure 1:**
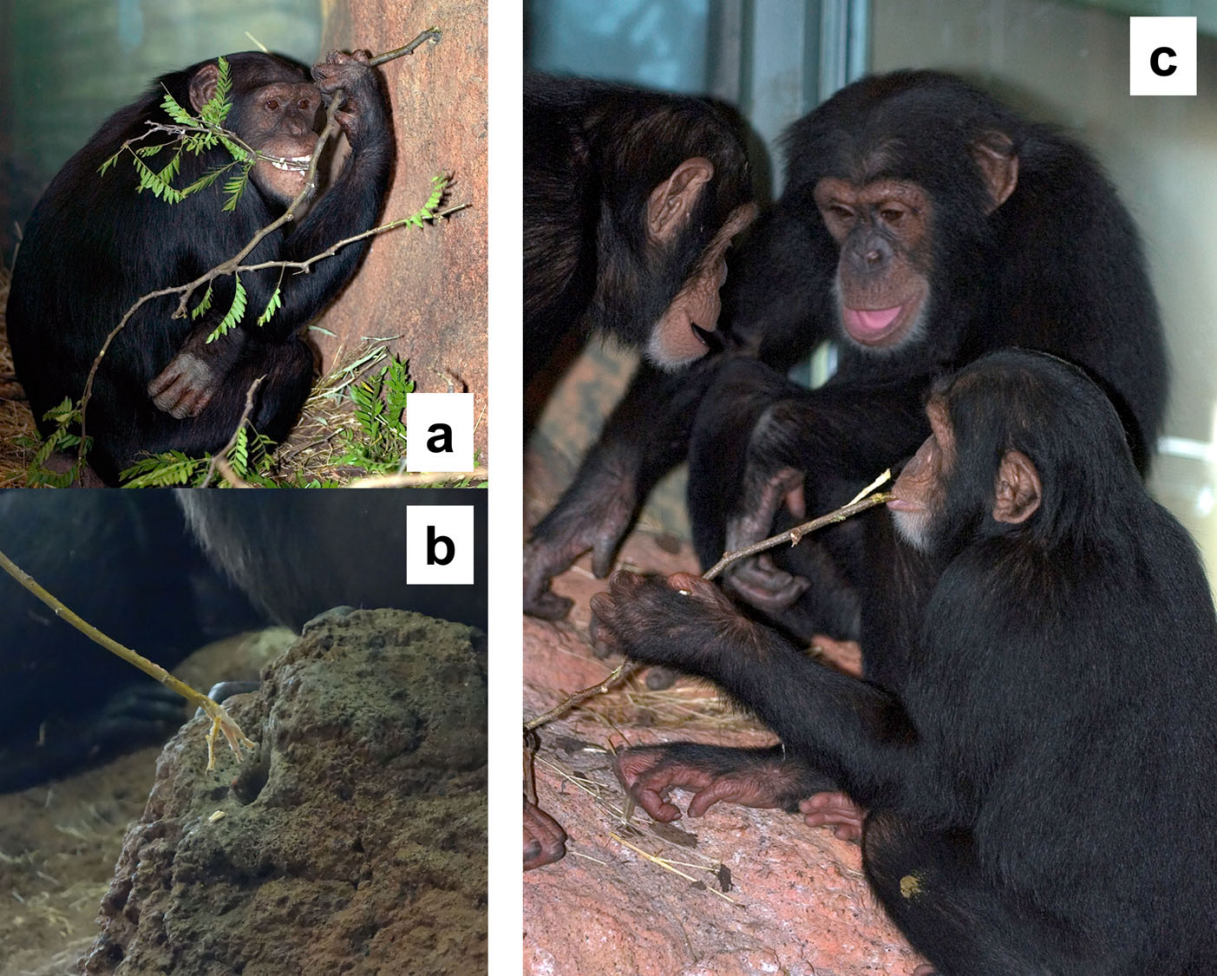
The artificial mound and tool-probing behavior of the chimpanzees at Lincoln Park Zoo, Chicago, showing (a) a chimpanzee modifying a tool, and (b) a frayed tool used for fluid dipping by one of the chimpanzees, and (c) chimpanzees actively fluid dipping.

For this study, liquid foods were presented to the chimpanzees via an artificial termite mound, mimicking the look of a natural termite mound found in wild ape habitats (Fig. 1). The mound containing the liquid foodstuffs measured 274 cm wide by 205 cm tall and protruded 104 cm into the chimpanzees’ exhibit. Distributed evenly across the outer shell of the mound were eight holes, each of which terminated in the interior with screw attachments for polyvinyl chloride (PVC) tubes. These tubes (4.5 cm diameter, 13 cm length) were filled with viscous foodstuffs (e.g., ketchup) and attached from the inside of the mound. Each of the eight tubes was filled up to a depth of approximately 5 cm of the food. Once baited in this manner, the chimpanzees could access the food in these tubes from their enclosure by reaching into each hole with probe tools.

**Figure 2:**
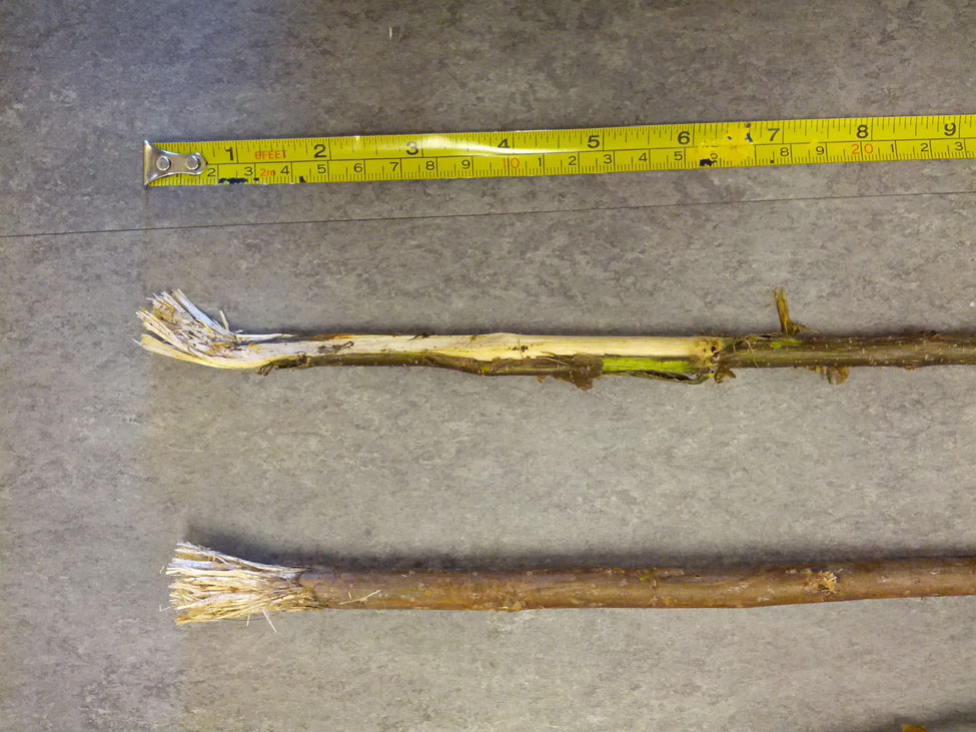
A serviceberry tree shoot (above) collected and modified for use as a probe tool by a chimpanzee. Also shown for comparison (below) is a poplar stick, provisioned to the chimpanzees by keeper staff, also modified for use a probe tool. NB: This photograph was taken of tools modified by this same group of chimpanzees in 2014, ten years after the data presented herein were collected.

Probe tools were not provided to the chimpanzees, but individuals were able to make tools from natural vegetation available in their indoor and outdoor exhibits [c.f. Lonsdorf et al., 2009; Tonooka et al., 1997]. Specifically, planted in their outdoor enclosure were several hawthorn trees (*Crataegus* spp.), serviceberry trees (*Amelanchier alnifolia*), red oak trees (*Quercus rubra*), and a bur oak tree (*Q. macrocapra*). Unfortunately, due to the degradation of the video footage collected in 2004 when these data were collected, for these current analyses, it was not possible to identify which species of plants were used by the chimpanzees to create their probe tools. However, contemporary observations of this group of chimpanzees revealed that they create tools from a number of species, including willow and poplar that are now provided to them by keeper staff, and they also use the young shoots of serviceberry trees that grow in their outdoor exhibit, which they modify by fraying the ends of the sticks (Fig. 2).

### Procedure

All data were collected over a 115 day period in 2004. The period when the termite mound was baited extended beyond this period but because the Baseline lasted for two months (see below), for this analysis we only include the chimpanzees’ responses in the first two months of the baited phase to enable a clearer comparison across the two periods. These data represent a subset of data previously collected at Lincoln Park Zoo as part of a wider investigation of ape social learning and tool use. The original findings from this study were published previously by Lonsdorf et al. [2009], but the analyses presented herein represent novel analyses and comparisons. Data are available upon request.

### Baseline

The first two months (56 days) of the study period represented the “Baseline” phase. Behavioral data were collected on 35 of these 56 days (i.e. Monday–Friday every week) from June 7th 2004–August 2nd 2004. These Baseline sessions were spread evenly throughout the Baseline period and all Baseline sessions were filmed for later analysis. During this period, the termite mound was never baited with food. The chimpanzees had no prior experience with obtaining food from this artificial mound and so this period represented a true behavioral baseline (see Lonsdorf et al. [2009] for full details of the chimpanzees’ background experiences).

### Experimental Phase: Bait and Control Sessions

On August 3rd 2004, the two month “Experimental” phase began and lasted 59 days (the last day was September 30th 2004). Spread evenly throughout the Experimental period were 19 “Bait” sessions, on which the tubes in the termite mound were baited with desirable viscous food (e.g. ketchup), at randomized times between 1100 and 1400 hr (Monday–Friday). No more than one Bait session was conducted on a given day. For Bait sessions, the chimpanzees did not know prior to testing what the termite mound was baited with, although, once baited, the chimpanzees could inspect the holes through smell and could also sample the food stuffs directly with probe tools [c.f. Bonnie et al., 2012; Finestone et al., 2014]. All Bait sessions were filmed for later analysis. On the remaining days within this period, the tubes were not baited and so represent “Control” days. Nineteen of the Control days were filmed and later coded for the analyses presented here (i.e. Control sessions).

### Coding and Analysis

Video footage of the chimpanzees’ behavior, on, or within 1 m of, the artificial termite mound, was recorded for 35 Baseline sessions, 19 Bait sessions, and 19 Control sessions. Video footage was recorded using a stationary, ceiling-mounted security camera which captured the entire mound and a 1 m perimeter around the mound. This camera was connected to a time-lapse VCR (Panasonic AG-RT650) that was pre-set to record each session. Data were scored directly from the video footage in 2004–2006, wherein each group member’s behavior was scored continuously, as described by Lonsdorf et al. [2009] (Table1). Video tapes were evaluated by four researchers that had been trained on the same protocol and who had passed an interobserver reliability test (≥85% agreement) before commencing scoring.

**I:** The Behavioral Ethogram Used to Code Chimpanzee Behavior

Behavior	Definition
Play	Individual may play by itself or with another individual. An individual may play with hands, fingers, and toes, other body parts, or an object may be handled and be the focus of play. The individual may be tossing, holding, wearing, carrying, chewing or making other contact with the object while making playful movements. May be either boisterous or quiet. May also include active play involving swing, dangling, leaping, somersaults, running, gamboling, pirouetting and bouncing. Social play is non-aggressive interactions involving two or more animals. Never accompanied by pilo-erection or agonism; may be accompanied by play-face and/or laughing.
Social Groom	Picking through hair or at skin of another individual and removing debris with hands and/or mouth. Includes licking self. Does not include pulling or plucking hair.
Other Prosocial	Individual engages in non-agonistic social behavior not defined elsewhere in the ethogram (i.e. kissing, embracing, sexual behavior, mother-infant behavior, holding hands).
Aggression	May include pilo-erection, and such behaviors as beating on or moving inanimate objects, stomping, slapping, swaying, hooting, chest-beat, lunge, rush, threats, wrestling, grab, bite, thrown, teeth baring, and clawing. This category will encompass all aggressive behaviors, whether directed at another individual or not.
Self-directed behavior	Picking through own hair or skin and removing debris with hand and/or mouth. Does not include pulling/plucking hair or scratching.
Feed/Forage	Individual is handling, manipulating or ingesting food items such as primate chow, biscuits, fruits, vegetables, natural vegetation, or enrichment. Includes foraging through bedding or other materials in search of desired food items.
Ride	Individual is clinging either ventrally or dorsally to another individual. Feet may not be on the ground/supportive surface.
Locomotion	Individual changes location in horizontal or vertical space by walking, running, crawling, etc. The change in location must be greater than one body length.
Inactive	Individual is not moving and not active in any other behaviors listed for 3 sec or more. Eyes may be open or closed.
Other	Individual engages in another behavior not listed in the ethogram (i.e. stealing bait from another individual’s tool, stealing another individual’s tool, non-aggressive push or taps).
Probe	Contact to the bait hole using a tool.
Poke	Poke or prod to the bait hole using fingers, no tool involved.
Investigate	Inspect the mound using visual or olfactory senses. No tool involved, no poking or prodding with fingers involved. Face must be oriented to the mound at a distance of less than 2 in for 3 sec or more.
Tool-modification	Interact with tool/tool-use material by modifying a tool, and otherwise manipulating tool-use material (i.e. pieces of the tool being broken off).
Interfere	Disrupting another individuals fishing by means of stealing the bait off their tool with either hands or mouth and/or stealing the tool itself.

The recorded data captured each session entirely but the data presented here represent the first 15 min of each session. For these analyses, we focused on the ‘probe’ and ‘tool-modification’ behaviors (Table1). Tool-modification behaviors incorporated both the manufacture and modification of probe tools, while probe described the chimpanzees actively using tools to procure liquid foods from the artificial mound. Although we have previously reported the behavior of this population of chimpanzees and their interaction with this artificial termite mound as ‘termite fishing’ [e.g. Lonsdorf et al., 2009], given that the mound was baited with liquid foods, we consider that the probing behaviors recorded during this study should be more accurately described as ‘fluid dip’ behaviors [c.f. Sanz & Morgan, 2009; though note that our underlying logic with regard to the usefulness of frayed tools remains the same in both cases].

As each of the three phases were comprised of a different number of test sessions (35, 19, and 19 respectively), all behaviors were converted to average rates/session. Friedman’s tests and Wilcoxon’s signed-ranks tests were used to compare the chimpanzees’ responses across conditions. Firstly, we compared the rate of fishing and tool-modification behaviors in the Baseline phase to (a) the days on which the termite mound was baited (Bait sessions) and (b) days within the experimental phase when the termite mound was not baited (Control sessions). Secondly, once the chimpanzees had experienced food in the artificial mound (Bait sessions), to determine whether tool modifications were functional, we compared the chimpanzees’ rates of tool modification and tool use across the Control and Bait sessions. To further investigate the interplay between tool-modification and probing behaviors, we ran a Pearson’s correlation between rates of tool modification and fishing behaviors. All analyses were conducted in IBM SPSS, Version 20 (IBM New York, NY).

We were also interested in determining if tool modification was more likely than other behaviors to be followed by tool usage (i.e. probing). For the purposes of this analysis, we simplified the continuous sampling dataset by noting whether or not, for each pair of behaviors observed, the first behavior was modification and whether or not the second behavior was tool use. All other behaviors described in Table1 (non-modification for the first, non-use for the second) were grouped into a single “other” category, creating a binary predictor variable and a binary response variable. We also wished to avoid pseudoreplication and account for systematic variation among individuals and training sessions, and thus included “chimpanzee” and “session” as random effects in our analyses. Finally, we were interested in determining if the relationship between modification and use differed systematically among individuals or sessions (i.e. if there was an interaction between the fixed effect and either random effect). Given the binary response variable and the combination of fixed and random predictor variables, we analyzed our data using a generalized linear mixed effects model. We fit this model using the Laplace approximation via the glmer function in the lme4 package [Bates et al., 2012] in R [R Development Core Team, 2010]. Model evaluation and simplification proceeded using *Z*-test and likelihood ratio tests (LRTs) [Bates et al., 2012].

## RESULTS

**Figure 3:**
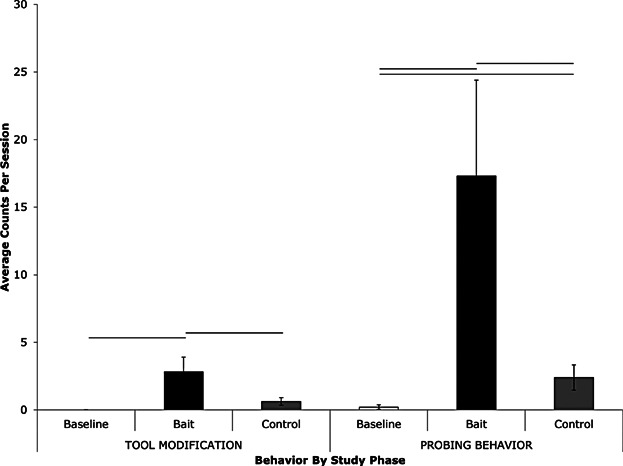
Average rate of tool-modification and tool probing behavior in the baseline, control, and bait sessions. Error bars show the +/− standard error of the mean. Horizontal lines indicate significant differences (*P* < 0.05).

There were significant differences across all three phases (Baseline, Bait, and Control) regarding both the rate of tool-modification behaviors as well as the rate of probing behaviors recorded (Fig. 3) and we consider each in turn. We then present analysis of the interplay between these behaviors.

### Tool Modification

There was a significant difference in the rate of tool-modification behaviors observed across the three experimental phases (Friedman’s test: *X*^2^(2) =* *11.273, *P* = 0.04). The chimpanzees performed more tool modification when the termite mound was baited (Bait) than during the Baseline (Wilcoxon’s signed-ranks test, *T*+ = 21.0, *N* = 7, *P* = 0.028) or Control (*T*+ = 0.00, *N* = 7, *P* = 0.028) sessions. While none of the chimpanzees ever modified tools in the Baseline sessions, six of the seven chimpanzees performed one or more tool-modification behaviors during the Bait phase (the alpha male, aged 14 years, was never observed to modify his tools) and all did so on the first Bait session (the first chimpanzee to modify a tool was a five-year-old male). Emphasizing the low rate of tool-modification behaviors in Control sessions, there was no difference in the number of tool-modification behaviors seen during the Baseline and Control sessions, but they showed a trend towards performing more tool modification in the Control sessions (*T*+ = 0.00, *N* = 7, *P* = 0.068, Fig. 3).

### Probing

During the Baseline sessions, five of the seven chimpanzees performed one or more probing behaviors and the first chimpanzee to do so was a five-year-old female during the 3rd Baseline session (June 10th 2004). In contrast, during the Bait sessions, all seven chimpanzees probed the artificial mound (six did so in the first session alone). There was a significant difference in the rate of probing behaviors observed across the three experimental phases (Friedman’s test: *X*^2^(2) = 14.00, *P* = 0.001). Comparable with their tool-modification behaviors, the chimpanzees performed more probing behaviors when the termite mound was baited (Bait) than during the Baseline (Wilcoxon’s signed-ranks test, *T*+ = 28.0, *N* = 7, *P* = 0.018) or Control (*T+* = 0.00, *N* = 7, *P* = 0.018) sessions. The chimpanzees were observed to probe more during the Control sessions compared to during Baseline sessions (*T*+ = 0.00, *N* = 7, *P* = 0.018), indicating comparably low levels of probing behaviors in both phases.

### Evaluating the Relationship Between Tool-modification and Probing Behaviors

Chimpanzees were only recorded to modify tools in Bait and Control sessions. During the Bait sessions, there was a significant positive correlation between rates of probing and tool-modification behaviors (Pearson’s *r* = 0.85, *N* = 7, *P* = 0.04), such that those chimpanzees that probed more, also performed more tool-modification behaviors. There was no significant correlation, however, between rates of probing behavior and tool modification in Control sessions (Pearson’s *r* = 0.66, *N* = 7, *P* = 0.108).

Overall, probing behaviors comprised 24.1% of the total behaviors observed. The rate of probing behaviors varied significantly among both chimpanzees (LRT, *X*^2^(1) = 862.32, *P* < 0.0001) and sessions (LRT, *X*^2^(1) = 91.61, *P* < 0.0001), although among-individual variance was substantially higher than among-session variance (1.62 and 0.14, respectively). After accounting for this variation, the fitted values revealed that probing behaviors occurred more frequently after modification (47.6%) than after other behaviors (14.9%), and this difference was statistically significant (*Z*-value: 12.92, *P* < 0.0001). Indeed, in Bait sessions, the average delay between a chimpanzee modifying a tool and then performing a probing behavior was only 4.5 sec (range = 1–247 sec). The degree to which modification predicted probing did not significantly differ among chimpanzees or sessions (LRTs, both *P* > 0.4).

## DISCUSSION

Through this study, we aimed to determine whether a group of chimpanzees modified probing tools in order to extract viscous foods (akin to ‘fluid dip’) from a novel ‘artificial termite mound’ in their enclosure and, if so, to identify under what circumstances they modified tools for fluid dipping behaviors. Specifically, we wished to record whether the chimpanzees modified their tools prior to using them, and this is what we found. Expanding upon a previous study, which had investigated the learning of tool-use behavior by chimpanzees [Lonsdorf et al., 2009], our data focused on the specific elements of their tool-modification behaviors and suggest that the chimpanzees only modified their probe tools when it was functionally relevant to do so (i.e. when the artificial termite mound was baited with food). Furthermore, after modifying a tool, the chimpanzees’ most likely next act was to commence probing the baited artificial termite mound, and they did so within 4.5 sec on average. Thus, tool modification predicted tool use.

Although captive chimpanzees have been documented to create and modify functionally-relevant tools, these studies also report individual differences across chimpanzees as a function of age or rearing history [Bania et al., 2009]. Considering wild chimpanzees, unlike certain tool-use behaviors (e.g. fluid dip and ant dip), which have been reported to be habitual or customary in two or more wild chimpanzee communities [Whiten et al., 2001], tool modification, in the form of tool fraying, does not appear to be ubiquitous across individuals or groups. For example, only a limited number of tools used by chimpanzees at the Lopé Reserve, Gabon, have been reported as frayed [Tutin et al., 1995], while the majority of tools used by the chimpanzees at Gashaka Gumti National Park, Nigeria, show signs of fraying [Fowler & Sommer, 2007]. This suggests that such fraying is an active modification process practiced only by certain individuals. Similarly, while all seven chimpanzees in our study performed probing behaviors, we found great inter-individual differences in their proclivity to do so, and only six of the seven were recorded to modify their tools. However, of the six that modified their tools, we found no significant difference across individuals in the relationship between their tool-modification and tool-use behaviors; all chimpanzees were equally likely to probe after modifying a tool.

Of the six chimpanzees that modified their tools, four did so in Control sessions when no food was available, perhaps due to “carry over” effects from Bait sessions (i.e. because the chimpanzees experienced the mound baited the previous day, they were more likely to assume it would be baited again). Indeed, Price et al. [2009] noted that some chimpanzees in their study of chimpanzee tool use also modified tools when it was not functionally appropriate. Price et al. [2009] concluded that this could either be because tool modification was learned from observing a conspecific, and so the behavior became more ‘potent’, or because, having learned the behavior socially, the subjects had a reduced understanding of the causality of their actions. Without the inclusion of asocial pre-tests, it cannot be fully determined whether some, or even most, of the chimpanzees in our study required social learning to acquire tool-modification skills. Additionally, given that we only analyzed video footage of the chimpanzees’ behavior, on, or within 1 m of, the artificial termite mound, it is possible we did not code all instances of tool modification. However, regardless of the mode of acquisition of these tool-modification behaviors, or the mechanism that underlay their ability to modify tools and use them appropriately [Holzhaider et al., 2008; Teschke et al., 2013], it is notable that the chimpanzees modified their tools significantly more when the mound was baited with food and that tool modification most often preceded tool use than other behaviors.

Six subjects began modifying tools as soon as there was available food (in the first Bait session), and because these chimpanzees made their first tool modification within 60 sec of each other, it is most likely that these behaviors were already known, but only elicited when it was appropriate for them to do so. However, we might consider too that the chimpanzees were able to (asocially) learn tool-modification skills rapidly. We evaluate both of these proposals in turn. First, although the task of probing an artificial termite mound was novel to this group of chimpanzees, it is likely that they had prior experience with probing given that this is a near-universal behavior among chimpanzees [Whiten et al., 2001]. Therefore, it is most likely that the chimpanzees were familiar with the physical properties, or ‘affordances,’ of these materials [Huffman & Quiatt, 1986] and could modify them to create optimal probe tools [Byrne, 1998]. However, even if the chimpanzees already knew how to create and use probing tools, the chimpanzees still had to apply their skills to this novel task and only modify tools when it was relevant (i.e. when food was available). Second, if we consider the possibility that the chimpanzees had no prior experience of using probe tools, then we must conclude that at least one subject was able to asocially discover how to modify tools prior to using them.

If probe tool manufacture and modification is within chimpanzees’ ‘Zone of Latent Solutions’ [*sensu* Tennie et al., 2009], and not a skill that requires social learning, then other (captive) populations should have been recorded to have made similar discoveries. In a comparable study, Tonooka et al. [1997] presented a group of chimpanzees with tubes filled with orange juice to test whether the chimpanzees would be capable of learning how to obtain it. Ultimately, eight of the nine chimpanzees used tools to obtain the juice and they adopted 15 different kinds of tools over the course of the 31 sessions. In contrast to our study, in which six chimpanzees rapidly modified a tool, Tonooka et al. [1997] reported that it was only by the ninth session that a chimpanzee first made a tool from scratch. This delay is most likely explained by the fact that the chimpanzees were able to use their hands to obtain the juice; the use of tools was not essential. Using tools required the chimpanzees to use a new skill, despite already knowing one that gained them the reward, demonstrating that they were not ‘conservative’ in their learning strategy [*sensu* Hrubesch et al., 2009].

In our study, tool modification predicted tool use. Regardless of whether the subjects in our study already knew how to create, modify, and use probe tools, or if they had to acquire the skills at the start of the study, they all had to adapt and apply their skills to this novel task. This demonstrates their flexible foraging abilities. This is perhaps not surprising as, when presented with novel problem-solving tasks, chimpanzees [Hopper et al., 2014; Manrique et al., 2013; Tonooka et al., 1997; Yamamoto et al., 2013], like other species [e.g., *Pongo pygmaeus abelii*, Lehner et al., 2011; *Corvus moneduloides*, von Bayern et al., 2009], have been shown to build upon previously-learned behaviors in order to gain food rewards. The intent of the chimpanzees in the present study is further emphasized when we consider that they only created and modified tools when it was functionally relevant to do so (i.e. when food was available to obtain with probe tools) and thus they appeared to make these modifications intentionally.

We defined tool modification broadly, but a specific form of probe tool modification that is commonly reported is tool fraying. While even early studies of chimpanzee probing behavior reported chimpanzees’ proclivity to fray the end of probe tools [e.g., Teleki, 1974], it is unresolved whether such tool modifications are intentional or not [Byrne et al., 2013; Takemoto et al., 2005]. Whether modifications occur as a by-product of the probe tool manufacture or use (i.e. chewing on the end of the tool to remove the food), or if it is done proactively in order to improve the functionality of the probe tool is still debated [Fowler & Sommer, 2007; Johnson, 2010]. For example, the frayed ends of honey collection tools could either be produced by a chimpanzee actively fraying the end of the tool or be created unintentionally if the chimpanzee broke the stick ‘progressively’, rather than forcefully [Boesch et al., 2009]. We propose that, in addition to looking at individual differences in chimpanzees’ proclivity to modify tools through fraying [Fowler & Sommer, 2007; Tutin et al., 1995], future observations of captive chimpanzees can also allow for detailed analysis of the sequence of fraying and tool use to determine whether it is a by-product of tool use or not [c.f. Sanz et al., 2009] and that studies with captive chimpanzees may offer a more controlled environment for such investigations.
